# ﻿*Opistognathusctenion* (Perciformes, Opistognathidae): a new jawfish from southern Japan

**DOI:** 10.3897/zookeys.1179.109813

**Published:** 2023-09-14

**Authors:** Kyoji Fujiwara, Hiroyuki Motomura, Gento Shinohara

**Affiliations:** 1 National Museum of Nature and Science, 4-1-1 Amakubo, Tsukuba, Ibaraki 305-0005, Japan National Museum of Nature and Science Tsukuba Japan; 2 The Kagoshima University Museum, 1-21-30 Korimoto, Kagoshima 890-0065, Japan The Kagoshima University Museum Kagoshima Japan

**Keywords:** Actinopterygii, dredge, new species, Osumi Islands, Ryukyu Islands, taxonomy

## Abstract

*Opistognathusctenion***sp. nov.** (Perciformes: Opistognathidae) is described on the basis of three specimens (17.3–30.6 mm in standard length) collected from the Osumi and Ryukyu islands, southern Japan in depths of 35–57 m. Although most similar to *Opistognathustriops*, recently described from Tonga and Vanuatu, the new species differs in mandibular pore arrangement, dorsal- and caudal-fin coloration, fewer gill rakers, and lacks blotches or stripes on the snout, suborbital region and both jaws.

## ﻿Introduction

*Opistognathus* Cuvier, 1816 is the most speciose genus of jawfishes (Perciformes: Opistognathidae), being distributed worldwide in tropical and temperate regions, except for the eastern Atlantic Ocean and Mediterranean Sea (Smith-Vaniz 2023); most species of *Opistognathus* occur in the Indo-West Pacific. A recent review of the genus by Smith-Vaniz (2023) recognized 60 valid species, 18 being new, and additional new species of *Opistognathus* were predicted. To date, valid species of *Opistognathus* total 91 overall (Smith-Vaniz 2023).

Examination of specimens in the Kagoshima University Museum, Japan (KAUM) and the National Museum of Nature and Science, Japan (NSMT) revealed an unidentified species of *Opistognathus*, collected in 35–57 m depth off the Osumi and Ryukyu islands, southern Japan. In common with the majority of species of *Opistognathus*, the number of known examples of the present species is small, due to difficulties in collecting, attributed to their small body size and cryptic habitat [for details see Smith-Vaniz (2023)]. Notwithstanding, the species is clearly distinct, having a unique combination of meristic characters and fresh coloration, and is here formally described as a new to science.

## ﻿Material and methods

### ﻿Morphological observation

Counts and measurements followed Smith-Vaniz (2023). Standard length (SL) was measured to the nearest 0.1 mm. Other measurements were made to the nearest 0.01 mm using needle-point calipers under a dissecting microscope (ZEISS Stemi DV4). Counts of vertebrae and fin rays, plus dorsal- and anal-fin pterygiophores, were examined from radiographs. Further osteological characters were investigated by computed tomography (CT) scanning using inspeXio SMX-225CR FPD HR Plus (Shimadzu, Kyoto) at 100 kV and 120 μA at a resolution of 18 μm, and three-dimensional reconstruction images produced by the rendering software VGSTUDIO MAX ver. 3.3 (Volume Graphics, Nagoya).

### ﻿Preparation of figures

Photographs of preserved specimens were taken with a Nikon D850 camera using an internal focus bracketing function; sets of multifocal images were then collated into a composite image, using Adobe Photoshop. The distribution map was prepared using GMT ver. 5.3.1, with data from GSHHG (Wessel and Smith 1996). The names and grouping of islands in southern Japan (belonging to Kagoshima and Okinawa prefectures) follow Motomura and Matsunuma (2022: fig. 5.2).

### ﻿Comparative data

Morphological characters of comparative species of *Opistognathus* are cited from Smith-Vaniz (2023).

## ﻿Results and discussion

### ﻿ Opistognathus ctenion sp. nov.

Taxon classificationAnimaliaPerciformesOpistognathidae

0E8E520F-A681-5EB6-BDB9-CFCAF43222F0

https://zoobank.org/66D79DFB-6CAA-4E18-A766-B2F117333C13

**Figure 1.**
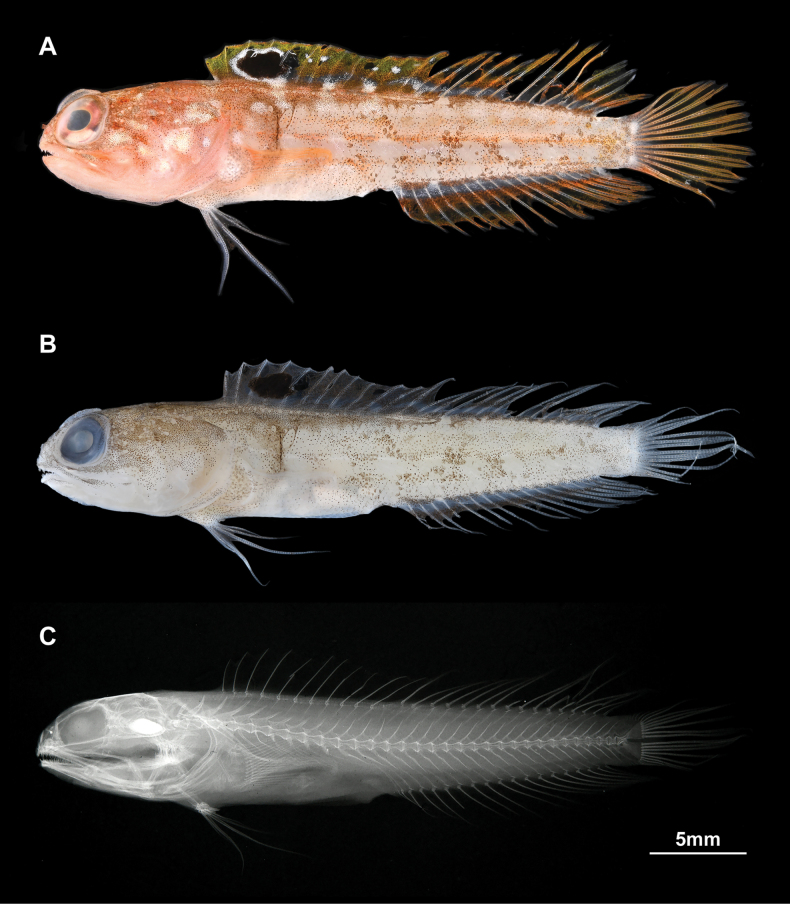
Holotype of *Opistognathusctenion* (KAUM–I. 174226, 30.6 mm SL, off Mage-shima island, Osumi Islands, Kagoshima, Japan) **A** fresh and **B** preserved specimens photographed by KAUM and K. Fujiwara, respectively **C** X-ray image, photographed by K. Fujiwara.

**Figure 2.**
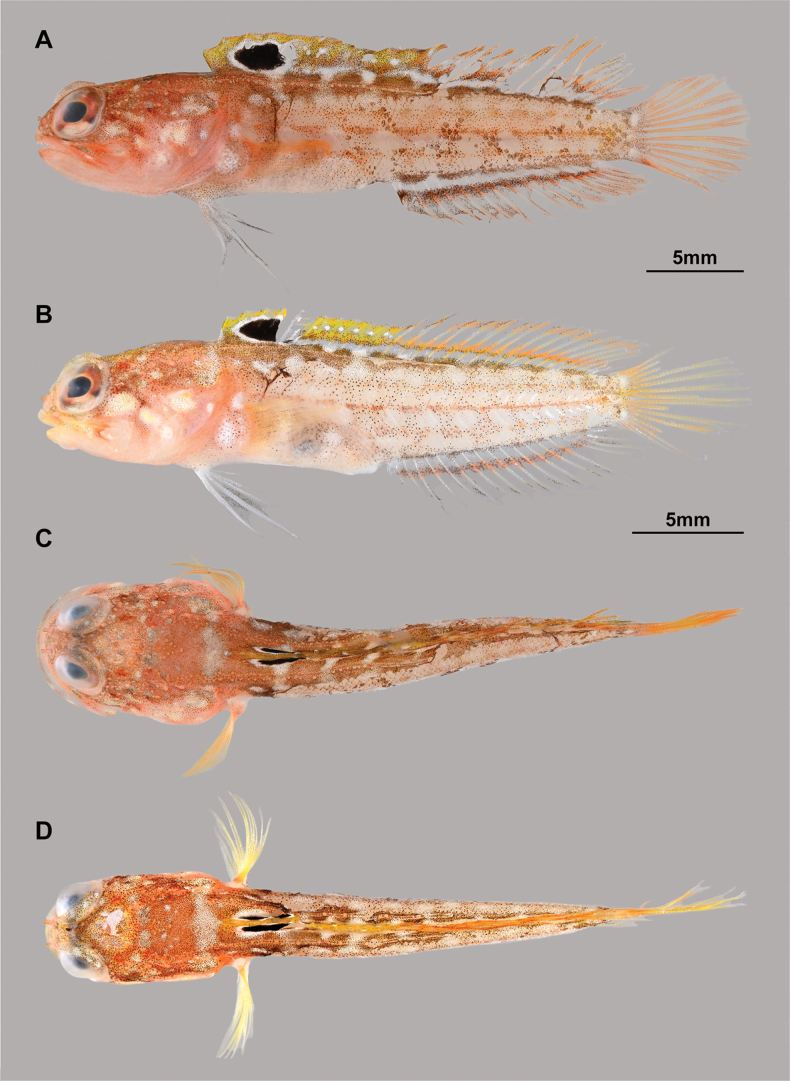
Fresh coloration of two paratypes (**A, C**KAUM–I. 174226, 30.6 mm SL **B, D**KAUM–I. 174227, 26.2 mm SL) of *Opistognathusctenion*, photographed by KAUM**A, B** lateral views **C, D** dorsal views.

**Figure 3.**
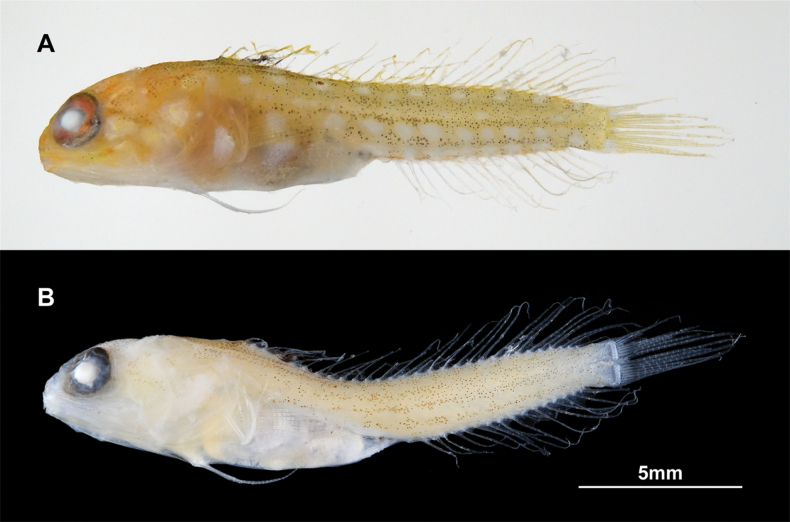
Small paratype of *Opistognathusctenion* (NSMT-P 130174, 17.3 mm SL) **A** fresh and **B** preserved specimens, photographed by G. Shinohara and K. Fujiwara, respectively.

**Figure 4.**
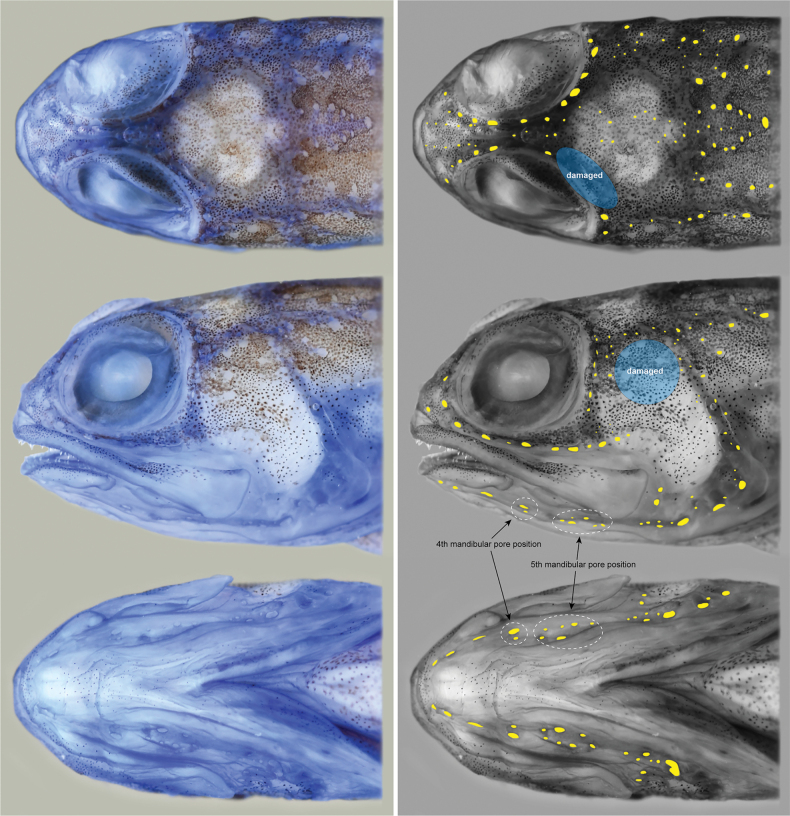
Head of holotype of *Opistognathusctenion* (KAUM–I. 174226, 30.6 mm SL), showing cephalic sensory pores (left column cyanine blue stain; right column solid yellow). Photographed by K. Fujiwara.

**Figure 5.**
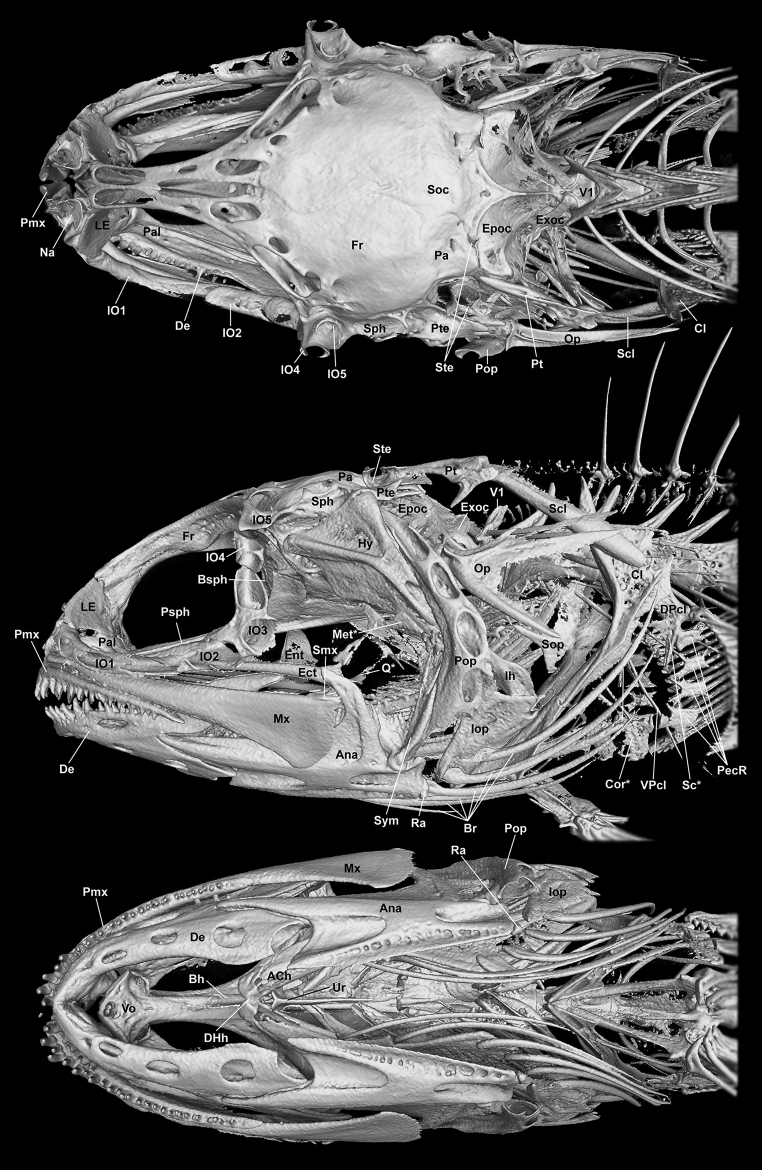
Three-dimensional reconstruction of head and anterior body in *Opistognathusctenion* (KAUM–I. 174226, 30.6 mm SL), based on CT scanning. Photographed by G. Shinohara and K. Fujiwara. Abbreviations: ACh, anterior ceratohyal; Ana, anguloarticular; Bh, basihyal; Br, branchiostegal rays; Bsph, basisphenoid; Cl, cleithrum; Cor, coracoid; De, dentary; DHh, dorsal hypohyal; DPcl, dorsal postcleithrum; Ect, ectopterygoid; Ent, entopterygoid; Epoc, epiotic; Exoc, exoccipital; Fr, frontal; Hy, hyomandibular; Ih, interhyal, IO1 to IO5, 1^st^ to 5^th^ infraorbitals, respectively; Iop, interopercle; LE, lateral ethmoid; Met, metapterygoid; Mx, maxilla; Na, nasal; Op, opercle; Pa, parietal; Pal, palatine; PecR, pectoral radial; Pmx, premaxilla; Pop, preopercle; Psph, parasphenoid; Pt, posttemporal; Pte, pterotic; Q, quadrate; Ra, retroarticular; Sc, scapula; Scl, supracleithrum; Smx, supramaxilla; Soc, supraoccipital; Sop, subopercle; Sph, sphenotic; Ste, supratemporal; Sym, symplectic; Ur, urohyal; V1, 1^st^ vertebral centrum; Vo, vomer; and VPcl, ventral postcleithrum. Asterisks indicate poorly resolved features.

**Figure 6.**
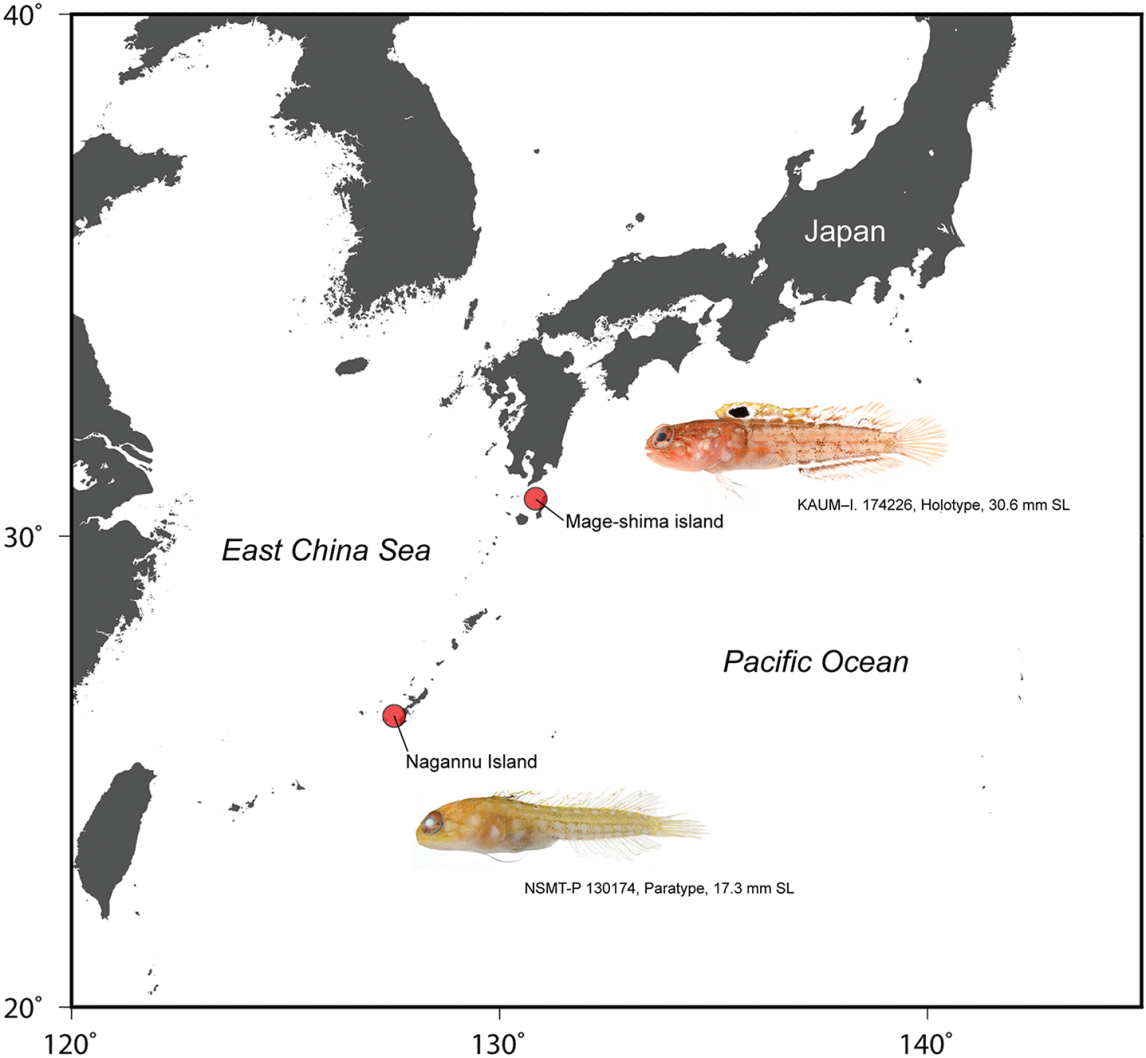
Distributional records of *Opistognathusctenion*.

Figs 1 , 2 , 3 , 4 , 5 , 6 ; Table 1 New English name: Japanese Whitespotted Jawfish New standard Japanese name: Shiratama-agoamadai

#### Type material.

***Holotype*.**KAUM–I. 174226, 30.6 mm SL, off Mage-shima Island, Osumi Islands, Kagoshima, Japan, 35 m depth, dredge, 29 Sept. 2022, K. Kubota. ***Paratypes*.**KAUM–I. 174227, 26.2 mm SL, collected with holotype; NSMT-P 130174, 17.3 mm SL, southwest of Nagannu Island, Kerama Islands, southern Ryukyu Islands, Okinawa, Japan (26°14′33"N, 127°31′19"E–26°14′30"N, 127°31′24"E), 53–57 m depth, dredge operated by R/V Toyoshio-maru (Hiroshima University), 19 May 2017, G. Shinohara.

#### Diagnosis.

A species of *Opistognathus* distinguished from congeners by the following combination of characters: posterior end of upper jaw rigid, without flexible lamina; dorsal-fin rays XI, 16–18; anterior dorsal-fin spines very stout and straight, and their distal ends not transversely forked; anal-fin rays II, 17; gill rakers 6 or 7 + 13 or 14 = 20 or 21; vertebrae 10 + 22 = 32; longitudinal scale rows c. 40–50; lateral line terminating below 4^th^–6^th^ soft ray of dorsal fin; 4^th^ and 5^th^ mandibular pore positions usually included 2 and 6–7 pores, respectively; body scales absent anterior to vertical below 4^th^ or 5^th^ dorsal-fin spine; vomerine teeth 2; body reddish-brown with 3 or 4 longitudinal rows of c. 8–10 whitish blotches; cheek and opercle with five or six whitish blotches; snout, suborbital region, and both jaws without blotches or stripes; spinous dorsal fin with ocellus between 2^nd^ to 5^th^ spines; dorsal-fin soft-rayed portion with two reddish-orange stripes; pectoral-fin base with one or two whitish blotches; caudal fin uniformly faint orange or reddish-yellow.

#### Description.

General appearance of type specimens as in Figs 1, 2 and 3. Lateral line system and osteological features of the holotype are given in Figs 4 and 5, respectively. Lateral line system and scale descriptions based on KAUM–I. 174226, 174227 (not available for NSMT-P 130174 due to poor specimen condition). Counts and measurements of type specimens are given in Table 1.

**Table 1.** Counts and measurements of *Opistognathusctenion*.

	Holotype	Paratype	Paratype
	KAUM–I. 174226	KAUM–I. 174227	NSMT-P 130174
Standard length (mm; SL)	30.6	26.2	17.3
Counts			
Dorsal-fin rays	XI, 16	XI, 18	XI, 18
Anal-fin rays	II, 17	II, 17	II, 17
Total pectoral-fin rays	19 (left) / 19 (right)	19 / 19	19 / –
Pelvic-fin rays	I, 5	I, 5	I, 5
Procurrent caudal-fin rays	5 + 5	5 + 5	–
Branched caudal-fin rays	12	–	–
Segmented caudal-fin rays	8 + 8 = 16	8 + 8 = 16	8 + 8 = 16
Longitudinal scale rows	c. 40–50	c. 40–50	–
Vertebrae	10 + 22 = 32	10 + 22 = 32	10 + 22 = 32
Gill rakers	7 + 13 / 7 + 14 = 20 / 21	6 + 14 / 6 + 14 = 20 / 20	– / 7 + 14 = 21
Measurements (% SL)			
Pre-dorsal-fin length	32.3	32.5	35.1
Pre-anal-fin length	63.3	59.7	65.1
Dorsal-fin base length	62.9	63.5	59.8
Anal-fin base length	34.3	37.0	34.2
Pelvic-fin length	22.6	21.1	21.7
Caudal-fin length	20.9	23.2	22.2
Body depth	15.3	16.0	10.3
Caudal-peduncle depth	7.9	8.0	6.5
Head length	32.3	31.9	34.3
Postorbital length	19.8	20.5	19.1
Upper-jaw length	17.4	17.2	17.4
Postorbital-jaw length	6.8	5.5	4.3
Orbit diameter	10.0	10.5	11.2
As % of head length			
Postorbital length	61.3	64.2	55.6
Upper-jaw length	53.9	53.8	50.8
Postorbital-jaw length	21.2	17.3	12.5
Orbit diameter	30.9	32.8	32.7

– indicates no data due to poor condition.

***Head and body*.** Body elongate, compressed anteriorly, progressively more compressed posteriorly. Anus situated just before anal-fin origin. Head cylindrical, its profile rounded. Eyes somewhat large, located dorsolaterally. Anterior nostril a short membranous tube with a tiny tentacle on posterior rim, when depressed not reaching posterior nostril; situated about mid-way between posterior nostril and dorsal margin of upper lip. Posterior nostril opening elliptical. Mouth terminal, obliquely inclined anterodorsally, forming angle of c. 20° with body axis. Anterior tip of upper jaw slightly before vertical through lower-jaw tip. Posterior margins of preopercle and opercle indistinct, covered with skin and generally rounded with slightly elongated flap on upper part, respectively. Gill opening wide, its uppermost point slightly below horizontal through dorsal margin of orbit in lateral view.

***Lateral line system*.** Cephalic sensory pores moderately developed, covering most of head except for lower part of cheek and area adjacent to dorsal-fin origin. Mandibular pore positions 1 and 2 each with a single similarly-sized pore; position 3 with a single pore (largest size of mandibular pores); positions 4 and 5 with 1 (only left side of KAUM–I. 174227) or 2 and 6 or 7 pores, respectively. Lateral-line pores moderate, mostly in single series above and below embedded lateral-line tubes. Lateral line ending below 4^th^ (KAUM–I. 174226) or 6^th^ (KAUM–I. 174226) soft rays of dorsal-fin rays.

***Scales*.** Scales mostly missing, scaled area and scale counts estimated from scale pockets. Lateral surface of body and belly scaled, except above and slightly below lateral line, area anterior to vertical below 4^th^ (KAUM–I. 174227) or 5^th^ (KAUM–I. 174226) dorsal-fin spine, pectoral-fin base, and chest. Head region and bases of vertical fins completely naked.

***Fins*.** Dorsal fin moderately low, its profile relatively uniform except for anterior part and slightly notched junction of spinous and segmented rays; 1^st^ dorsal-fin spine distinctly short, its base located between uppermost point of gill opening and posteriormost tip of flap on opercle; all dorsal rays branched distally. Anal fin of similar height to dorsal fin, its origin vertically level with base of 1^st^ (KAUM–I. 174226) or 2^nd^ (KAUM–I. 174227, NSMT-P 130174) dorsal-fin soft ray; last anal-fin ray close to caudal-fin base and vertically level with last dorsal-fin ray; all fin rays branched distally. Pelvic-fin origin anterior to vertical through dorsal-fin origin; first ray of pelvic fin robust, not tightly bound to second ray; membrane between first and second rays incised distally; second ray longest, innermost 3 rays branched. Pectoral-fin base below 2^nd^ and 3^rd^ dorsal-fin spine bases. Caudal fin rounded posteriorly.

***Osteological features*.** Nasal short, tube-like. Vomer rhombic, with two tiny conical teeth anteriorly. Lateral ethmoid somewhat broad, articulating with 1^st^ infraorbital and palatine ventrally. Palatine robust anteriorly, tapering posteriorly, without teeth. Infraorbitals relatively slender, comprising 5 elements, including dermosphenotic; 1^st^ infraorbital longest, 3^rd^ with suborbital shelf, 5^th^ (= dermosphenotic) firmly attached to sphenotic. Basisphenoid crescentic. Frontal tapering anteriorly, 6 large dorsal openings for sensory canal from anteriormost tip to lateral aspect. Left and right parietals separated by supraoccipital. Anterior and posterior tips of supraoccipital strongly pointed. Sphenotic not expanded. Supratemporals associated with parietal and pterotic.

Premaxilla with a single row of conical teeth, except for posterior end. Maxilla long, posteriorly broadly expanded with slightly rounded corners. Supramaxilla small, on upper posterior end of maxilla. Dentary with a single row of conical teeth; 5 large ventral openings (including on posterior tip) from mandibular sensory canal. Anguloarticular large, its anterior projection fitting into dentary notch; coronoid process strongly pointed, directed anterodorsally. Retroarticular small, on ventroposterior corner of anguloarticular. Hyomandibular broadly attached to sphenotic and pterotic. Ectopterygoid and symplectic slender. Entopterygoid forming a large shelf. Metapterygoid and quadrate present but poorly resolved, Opercle with 2 strong and 1 weak ridge. Preopercle with 5 large openings from preopercular sensory canal. Subopercle small, its anterior tip pointed. Interopercle triangular, size similar to subopercle. Six long recurved branchiostegal rays.

Posttemporal L-shaped, forked, dorsal limb articulating with epiotic, an opening on posterior corner. Supracleithrum rod-like. Cleithrum with a large dorsal blade, receiving supracleithrum. Dorsal postcleithrum rectangular, articulating with cleithrum and scapula. Ventral postcleithrum long, narrow. Scapula widely separated from coracoid. Pectoral-fin radials comprising 4 elements, lowermost distinctly largest. Supraneural bone absent. Anterior dorsal- and anal fin interdigitation patterns //1/1+1/1/ and //1+1/1/1/1/, respectively.

#### Coloration.

***Fresh coloration of holotype and KAUM–I. 174227*.** Head ground color reddish-brown dorsally, reddish-white ventrally. Iris generally reddish-brown, except for whitish area ventrally, with four faint dark red lines radiating from pupil. Two faint dark-red oblique lines, extending from just behind eye to middle of nape and upper part of cheek, respectively. Five or six whitish blotches on cheek and opercle. Floor of mouth entirely white No blotches or stripes on snout, suborbital region, and both jaws. Body reddish-brown, with 3 or 4 longitudinal rows of c. 8–10 whitish blotches of size distinctly smaller than blotches on head region; upper one or two rows and anterior part of lower two rows of blotches somewhat indistinct. Two whitish blotches on pectoral-fin base, lower blotch distinctly the larger. Dorsal- and anal-fin bases edged with dark reddish-brown, anterior edge of former extending slightly below lateral line (sometimes interrupted by body ground color). Spinous dorsal fin greenish- or yellowish-brown; an ocellus between 2^nd^ to 5^th^ spines, 4–6 white spots forming a longitudinal row just behind ocellus. Soft-rayed part of dorsal fin and anal fin hyaline or faint reddish-brown, with two and one reddish-orange stripes, respectively; upper stripe of former through distal edge, remaining stripes at c. 1/3 height of both fins. Pelvic-fin rays whitish and membrane hyaline with melanophores. Pectoral and caudal fins uniformly faint orange or reddish-yellow.

***Fresh coloration of NSMT-P 130174*.** Generally similar to other type specimens, with the following differences. Head and body yellow. Whitish blotches on body more distinct. A whitish blotch on pectoral-fin base. Vertical fins faintly yellow (details of pigmentation patterns not visible), an ocellus on spinous dorsal fin. Pelvic fins white.

***Color in alcohol*.** Head and body generally blackish-gray. Ventral part of head and belly white. Whitish blotches on cheek, opercle, and pectoral-fin base (in fresh condition) faded, traces of blotches on body represented by non-pigmented areas. Spinous dorsal fin generally blackish-gray, an ocellus apparent (with hyaline white edge), but longitudinal row of white spots faded. Soft-rayed part of dorsal and anal fins hyaline, reddish-orange stripes (in fresh condition) retained as blackish-gray stripes. Pelvic-fin rays white, membrane hyaline with melanophores. Pectoral and caudal fins uniformly translucent white.

#### Distribution and habitat.

Currently known only from the Osumi and Ryukyu islands, southern Japan in depths of 35–57 m (Fig. 6). The Ryukyu specimen (NSMT-P 130174) was collected from a sandy gravel bottom.

#### Etymology.

The specific name is a noun in apposition derived from the Greek diminutive κτενίον, meaning “a small comb”. It refers to the low gill raker numbers in the new species, one of the lowest recorded for Indo-Pacific species of *Opistognathus* (see below).

#### Comparisons.

*Opistognathusctenion* keys out to couplet 25 in Smith-Vaniz’s (2023) key to species of *Opistognathus* (including all valid species known from the Indo-West Pacific to date). The new species is most similar to the allopatric *Opistognathustriops* Smith-Vaniz, 2023 in having the following characters: posterior end of upper jaw rigid, without flexible lamina; dorsal-fin rays XI, 16–18; anal-fin rays II, 17; vertebrae 10 + 22 = 32; longitudinal scale rows c. 40–50; body scales absent anterior to vertical below 4^th^ or 5^th^ dorsal-fin spine; vomerine teeth 2; lateral line terminating below 4^th^–6^th^ soft ray of dorsal fin; and spinous dorsal fin with an ocellus between 2^nd^ to 5^th^ spines. However, *O.ctenion* differs distinctly from *O.triops* in having fewer gill rakers (6 or 7 + 13 or 14 = 20 or 21 in *O.ctenion* vs 8 or 9 + 16–18 = 24–27 in *O.triops*), usually 2 and 6 or 7 pores included in the 4^th^ and 5^th^ mandibular pore positions, respectively (vs 1 and 2–4 pores, respectively), two reddish-orange stripes on the soft-rayed part of the dorsal fin (vs three broken brown stripes), a uniformly faint orange or reddish-yellow caudal fin (vs hyaline with three brown bars), and no blotches or stripes on the snout, suborbital region, and both jaws (vs 4 or 5 brown lines radiating from orbit). In addition, *O.ctenion* apparently occupies a slightly deeper water habitat than *O.triops* (currently known from 35–57 m depth vs 12–32 m depth).

The total of 20 or 21 gill rakers in *O.ctenion* is one of the lowest among the Indo-Pacific species of *Opistognathus*, with only two species sharing similar counts [viz., *Opistognathusalbomaculatus* Smith-Vaniz, 2023 with 19–22 gill rakers; and *Opistognathusreticulatus* (McKay, 1969) with 21–23; see Smith-Vaniz (2023: table 12)]. Although *O.ctenion* is unlikely to be misidentified as *O.reticulatus* due to significant differences in body color, it is somewhat similar to *O.albomaculatus* in sharing whitish blotches on the body. However, the former can be easily distinguished from *O.albomaculatus* by the ocellus on the spinous dorsal fin (vs a striped pattern in *O.albomaculatus*). Dorsal- and anal-fin ray, and caudal vertebral numbers, as well as vomerine teeth condition, are also useful for distinguishing between the two species (viz., XI, 16–18 and II, 17, respectively in *O.ctenion* vs X, 19–21 and II, 18–20 in *O.albomaculatus*; 22 vs 23–25; and two teeth present vs teeth absent).

## Supplementary Material

XML Treatment for Opistognathus ctenion

## ﻿Additional information

### Author ORCIDs

Kyoji Fujiwara https://orcid.org/0000-0001-7577-8333

Hiroyuki Motomura https://orcid.org/0000-0002-7448-2482

Gento Shinohara https://orcid.org/0000-0002-8071-9239

### Data availability

All of the data that support the findings of this study are available in the main text.
